# Virtual reality video promotes effectiveness in advance care planning

**DOI:** 10.1186/s12904-020-00634-w

**Published:** 2020-08-16

**Authors:** Wan-Ting Hsieh

**Affiliations:** Department of Palliative Medicine, Chi-Mei Medical Center, No. 901, Zhonghua Rd., Tainan, 710 Taiwan

**Keywords:** Virtual reality video, Decision tool, Advance care planning, Advance decision, End-of-life care, Patient autonomy act

## Abstract

**Background:**

In 2019, the Patient Autonomy Act went into effect, allowing Taiwanese citizens to establish legal advance decisions. In an effort to secure a more realistic and accurate perception of situations, a virtual reality video was developed by the palliative care team of Chi-Mei hospital in southern Taiwan for citizens to use before advance care planning. This study explores the change in participants’ preference and certainty regarding end-of-life decisions after using this tool.

**Methods:**

Participants were at least 20 years old and capable of reading and understanding the information provided in the written handout with information about the legal process of making an advance decision. They completed pre-test questionnaires, viewed a six-minute 360-degree virtual reality video on a portable headset, and then completed a post-test questionnaire about their preference on the five medical options—CPR, life-sustaining treatments, antibiotics, blood transfusion, and artificial nutrition and hydration—followed by feedback on the helpfulness of the virtual reality. The control group included 40 participants who only read the handout and completed pre-test and post-test questionnaires.

**Results:**

After viewing the virtual reality video, preference for not using CPR, life-sustaining treatment, antibiotics, blood transfusion, and artificial nutrition and hydration increased significantly in the virtual reality intervention group. Uncertainty regarding the five medical options mentioned above significantly decreased. The intervention was generally recognized by participants for its help in making decisions.

**Discussion:**

The decrease in the number of participants who could not make decisions indicates that the virtual reality video may be helpful for users in making end-of-life decision. According to feedback, the virtual reality video helped equip users with better understanding of medical scenarios, and that it is a good decision tool for advance care planning.

**Conclusion:**

This is the first study since the Patient Autonomy Act has been passed that explores the effectiveness of using a virtual reality video as a decision tool in advance care planning and reveals decreased preference of CPR, life sustaining treatment, antibiotics, blood transfusion, and artificial nutrition and hydration after intervention. This decision aid proved to be an effective tool for clarifying their end-of-life care preferences.

## Background

In January 2019, the Patient Autonomy Act of Taiwan (hereafter, “the Act”), the first of its kind in Asia, officially went into effect, allowing Taiwanese citizens to establish a legally binding advance decision (AD). An AD enables someone to refuse specified medical treatment in the future when they may lack the capacity to consent to or refuse medical treatment [1]. According to the Act, a patient must complete an advance care planning (ACP) consultation with a medical team before documenting their wishes with a formal AD. The aim of this consultation is to ensure that the wishes, values, and preferences of a patient concerning future care and treatment are documented in their AD and will be respected when needed [2, 3].

Our hospital (a medical center in southern Taiwan) is one of the seven trial hospitals entrusted by the Ministry of Health and Welfare for the implementation of the Act since it was passed at the end of 2015 and announced in January 2016 (a three-year preparation period accompanied the Act). Observations suggest that Taiwan citizens had a strong desire for self-determination in end-of-life medical decisions but were limited by insufficient knowledge of medical treatments, clinical scenarios, education, and legal literacy. There is a great deal of clinical knowledge and legal terms that have to be explained and presented in a way that the general public can understand. In the past, medical options have typically been written on handout forms provided to patients with a short conversation between medical teams and patients before patients made their choices. However, imagining medical treatments and future disease status merely based on reading materials and verbal communication is likely insufficient for patients to truly clarify their preferences for end-of-life decisions [4] To improve this situation, several video decision tools have been introduced to ACP to improve the accuracy and certainty of end-of-life decision making, and have been found valuable in helping patients increase their knowledge of medical treatments and allow them to clarify their medical decision preferences with more certainty [5–7].

A previous neuroscience study has shown that video decision tools enable patients to be more informed and confident about their medical decision making, because the visual cortex under video-watching prompts the brain in decision making [8]. We developed a virtual reality (VR) video in 2017 to supplement the standard way of decision making before ACP and to determine more realistic and accurate perceptions of patients as they explore their values and preferences for end-of-life treatment. Virtual reality (VR) is a computer technology that provides users with a simulated and immersive experience. The lifelike and realistic environment of VR can facilitate emotional responses [9]. The impact of emotion in decision making is well-recognized [10, 11]. VR may make it easier for users to understand aspects of life-sustaining treatment (LST), and to arouse emotions that will make their decision making more aligned with their true values. VR technology has been recognized by several studies as an effective tool in therapy for rehabilitation, clinical surgical training, pain management, and diagnostics [12–16]. However, none of the video decision tools for ACP are currently designed with VR technology. This research aims to apply VR video as a patient decision tool for ACP in order to supplement the traditional handout forms. In this study, we examined whether VR video can help users make end-of-life decisions and clarify their preferences by comparing their choices for end-of-life medical treatment before and after experiencing the VR video and collecting their feedback on the research experience.

## Methods

### Participants

This study had a total of 160 participants, 40 of whom were randomly assigned to the control group and 120 of whom were randomly assigned to the intervention group with the VR video. Participants were at least 20 years old and capable of reading and understanding the information provided in the written handout on the legal process of making an advance decision. The one who could read the words and understand the general healthcare knowledge were recognized as capable of reading and understanding. The reading and understanding capacity were assessed by the research assistants face to face. Recruitment occurred between January 23, 2019 and May 10, 2019 by flyer in a medical center, one long-term care facility, and activity centers in the community in Tainan, Taiwan. Eligibility criteria of participants included being over the age of twenty and capable of reading and verbally communicating. Written informed consent was obtained from all eligible participants after receiving explanations concerning the study’s purpose, methods, protection of anonymity, and freedom to withdraw. Institutional Review Boards from Chi-Mei Medical Center approved all study procedures (IRB approval number: 10710–008).

### Design

**Fig. 1:**
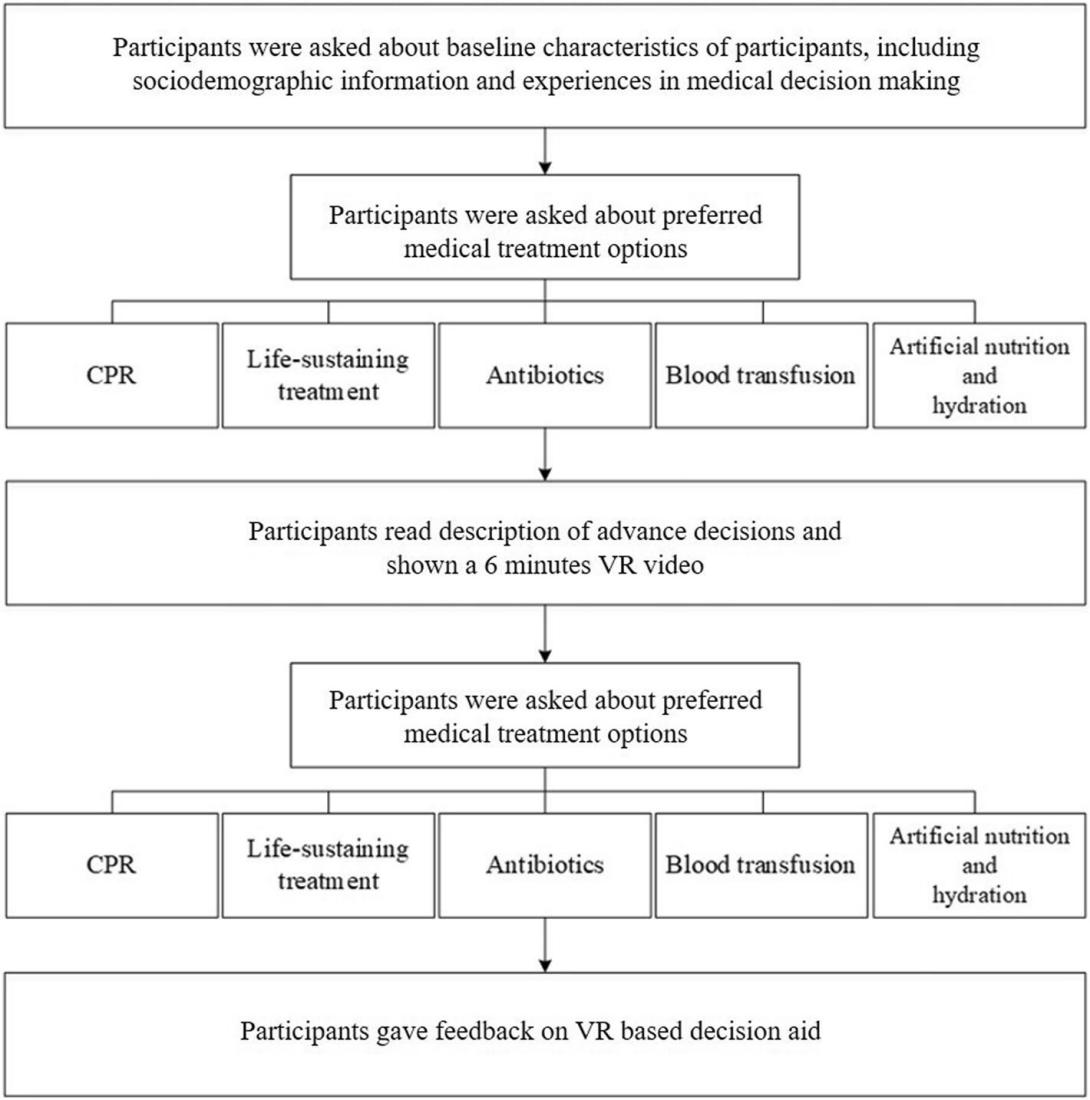
displays the flow chart of the whole research design. All participants answered pre-test questionnaires containing five choices about life-sustaining treatment options and artificial nutrition preference, read the handout, watched a 6-min VR video, and then answered a post-test questionnaire identical to the pre-test one

The language used in the handout and questionnaires was traditional Chinese. Participants were asked to complete written questionnaires about sociodemographic information, past experiences of medical decision-making, and preference for different kinds of treatment options for when they meet the clinical conditions prescribed by the Act, namely terminal illness, irreversible coma, permanent vegetative state, severe dementia, and other incurable acute and critical diseases that will be announced by theMinistry of Health and Welfarein the future. The sociodemographic data included gender, age, and level of education. The past experiences questionnaire included knowledge of AD and do not resuscitate orders (DNR), self-reported completion of DNR, experiences of caring for loved ones with terminal illness, and experiences of making medical decisions for them on whether to use life-sustaining treatment and artificial nutrition and hydration at that time. Five treatment options were included: CPR, life-sustaining treatment, antibiotics, blood transfusion, and artificial nutrition and hydration. Participants who were unable to decide whether to use treatments if they fell under specific clinical conditions, or were unconscious, or unable to clearly express their wishes could choose “uncertain.” The questions regarding individual preference for treatments were intended to observe differences among participants’ decisions after the intervention. The process of the VR video watching intervention group is shown in the diagram below (Fig. 1).

Participants were asked to read a handout published by Hospice Care Foundation of Taiwan with clear information including the introduction of the Act and an illustration of the legal process for making an effective AD to help the participants understand their fundamental right to make medical choices for themselves.

Participants then viewed a six-minute VR video that was produced by the palliative care team of our hospital on a portable VR headset. The 360-degree VR video was filmed and developed by members of the palliative care center with experts from different professional backgrounds including physicians, nurses, senior social workers, and psychologists to ensure the comprehensiveness of the presented clinical information and scenarios. Technical expertise was provided by a commercial company that specializes in VR techniques. The designed video used a first-person perspective of a patient with chronic obstructive pulmonary disease (COPD) to allow participants to immerse themselves in the complete clinical process of typical end-of-life care, starting with CPR in the intensive care unit, followed by withdrawn LST, hospice ward care, and hospice home care. The video also displayed the soul of this patient at the end of this film to reflect spiritual care. The displayed film features physicians, nurses, psychologists, and relatives, so that in addition to medical scenarios in different settings, the process involved consensus among family members.

Participants then completed the post-test questionnaire. Identical questions were reassessed regarding individual preference for treatment options when they are under specific clinical conditions, unconscious, or unable to clearly express wishes, followed by a feedback survey about the benefits of the decision tool using a 5-point Likert scale (strongly agree, agree, neutral, disagree, strongly disagree). The nine feedback questions were developed on the basis of research by Hossler et al. (2011) [17], and evaluated the effect of VR video on preparing oneself to open a discussion with doctors, family and others, choosing a spokesperson, clarifying one’s value and preference regarding medical treatments, equipping one’s understanding of medical scenarios and AD as well as making end-of-life decisions. Validity of the whole questionnaire was established by using a panel of experts including two palliative doctors, one oncologist, one judge, and one chair professor of the Chi-Mei medical center to review the wording, content, and constructs.

### Statistical analysis

Participants’ characteristics and past experiences in medical decision-making were described using descriptive statistics with frequency distributions. Participants’ feedback about the VR based decision tool after the intervention was summarized using means and standard deviation. The impact of the intervention change was evaluated with a one-sided McNemar’s exact test. Data were organized using SPSS 22 and analysis was performed using R software (version 3.6.0) and R package “exact2x2” (cite: Fay MP (2010). “Two-sided Exact Tests and Matching Confidence Intervals for Discrete Data.” R Journal, 2(1), 53–58. https://journal.r-project.org/.) The significance level was α = .05.

Post-hoc power analysis was performed for the primary aim of detecting the difference in uncertainty of the five medical options before and after VR intervention. A sample size of 120 in intervention group achieved 79% (96, 67, 80 and 94%) power to detect the difference that was reported in results section in uncertainty of using CPR (life-sustaining treatment, antibiotics, blood transfusion, and artificial nutrition and hydration), respectively before and after VR intervention using a one-sided McNemar test with a significance level of 0.05.

## Results

### Study participants

Baseline characteristics of participants are presented in Table 1.

**Table 1:** Baseline characteristics of participants

	Control group, *N* = 40 N(%)	Intervention group, *N* = 120 N(%)	*p*-value
Gender			0.3344
Male, 1	16 (40.00)	38 (31.67)	
Female, 2	24 (60.00)	82 (68.33)	
Age			0.7116
20–29	6 (15.00)	22 (18.33)	
30–39	13 (32.50)	33 (27.50)	
40–49	10 (25.00)	40 (33.33)	
50–59	9 (22.50)	18 (15.00)	
60≦	2 (5.00)	7 (5.83)	
Educational level			> 0.9999
Elementary school and below	0 (0.00)	2 (1.67)	
High school graduate	4 (10.00)	14 (11.67)	
College graduate and above	36 (90.00)	104 (86.67)	
Q5 Heard of DNR	32 (80%)	95 (79.17%)	0.9102
Q6 Signed a DNR permit	4 (10.00)	22 (18.33)	0.3219
Q7 Have heard about Patient Autonomy Act	33 (82.50)	100 (83.33)	0.9030
Q8 Have experience of caring for terminally ill loved one until death			0.7316
Yes (primary caregiver)	12 (30.00)	32 (26.67)	
Yes (not primary caregiver)	21 (52.50)	60 (50.00)	
No	7 (17.50)	28 (23.33)	
Q9 Have experience of LST decision making for loved one	5 (12.50)	17 (15.17)	0.7909
Q10 Have experience of ANH decision making for loved one	7 (17.50)	24 (20.00)	0.7290

DNR means the “Do Not Rescue Form,” a form signed by a patient or their closest relatives including the options about refusal of CPR and LST during the predying status under severe illness or injury.

### Preference for treatment options

**Fig. 2:**
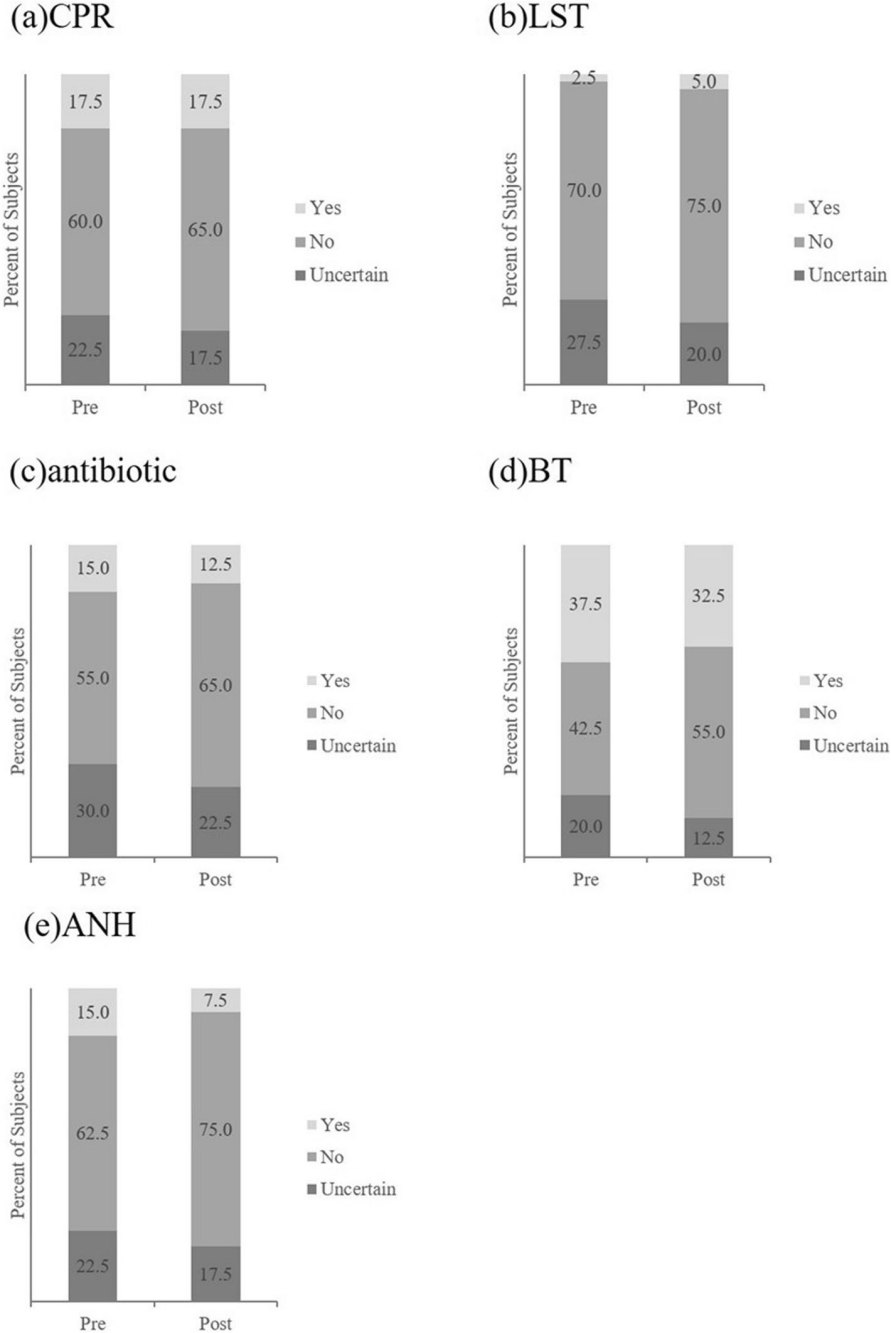
illustrates the control group’s change of the percentage of individual preference for the five medical treatments before and after participants read the handout and there is no significant statistical difference

**Fig. 3:**
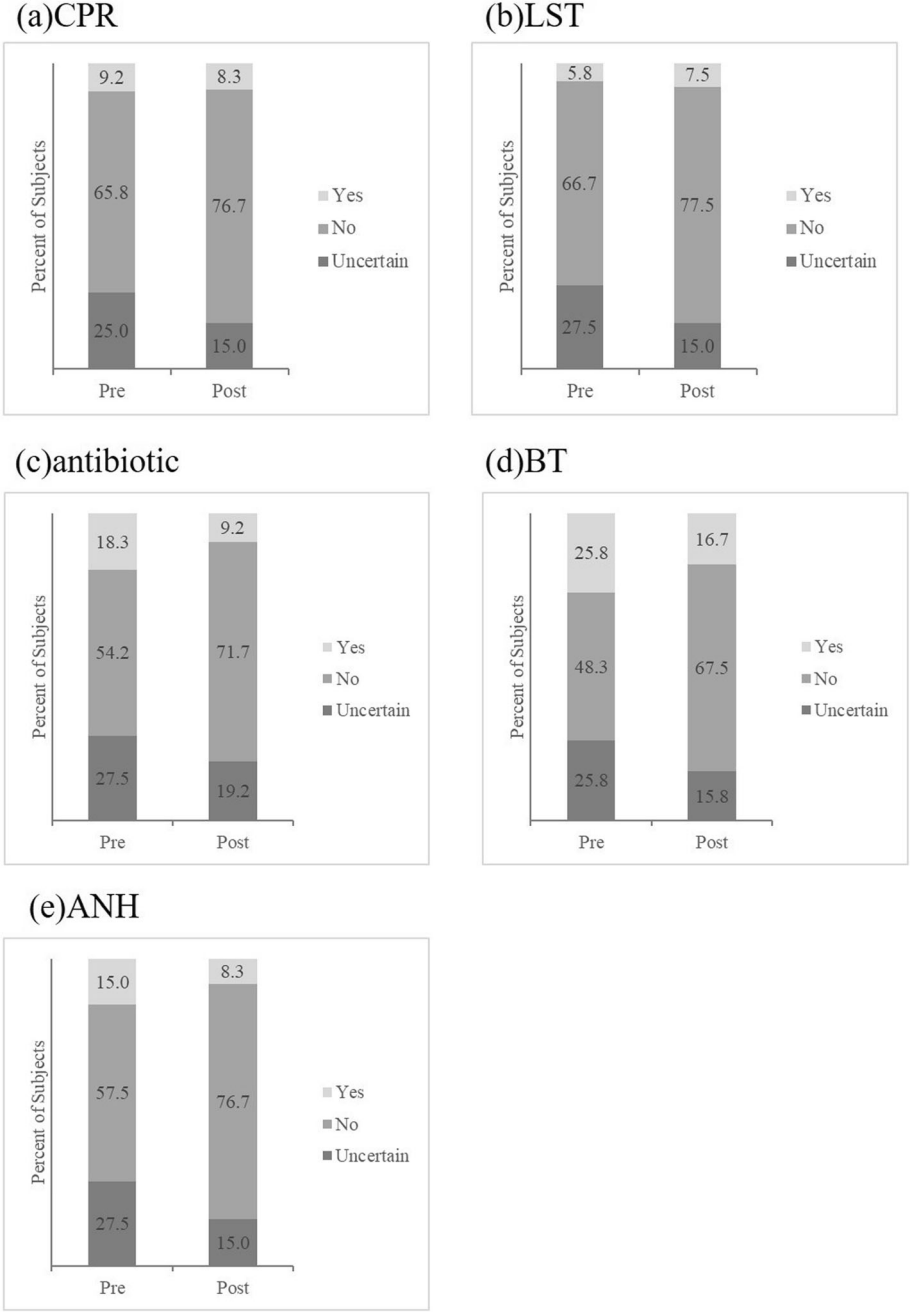
illustrates the results of the intervention group after participants read the handout and viewed the VR video. After the VR video intervention, preference to not use CPR, LST, antibiotics, blood transfusion (BT), and artificial nutrition and hydration (ANH) increased significantly. Uncertainty about using CPR, LST, antibiotics, blood transfusion, and artificial nutrition and hydration decreased significantly. This trend is not observed in the statistical analysis of the control group

Figure 2 illustrates the control group’s change in the percentage of individual preference for the five medical treatments before and after participants read the handout. Figure 3 illustrates the results of the intervention group after participants read the handout and viewed the VR video.

Table 2 shows the results of the statistical analysis for the pre-test questionnaires. After the VR video intervention, preference to refusal of CPR, LST, antibiotics, blood transfusion (BT), and artificial nutrition and hydration (ANH) increased significantly. Uncertainty about using CPR, LST, antibiotics, blood transfusion, and artificial nutrition and hydration decreased significantly. This trend was not observed in the statistical analysis of the control group.

**Table 2:** *P*-Values of Control and Intervention Groups

	Control group	Uncertain	Intervention group	Uncertain
	Refusal		Refusal	
**CPR**	0.25	0.25	0.00209	0.009605
**LST**	0.25	0.125	0.003769	0.0006561
**Antibiotic**	> 0.9999	0.125	< 0.0001	0.02069
**BT**	0.9688	0.1875	< 0.0001	0.00845
**ANH**	0.8906	0.3437	< 0.0001	0.001288

One-sided McNemar’s exact test was performed.

Control group participants read handout only.

Intervention group participants read handout and watched the VR video.

### Feedback on the VR decision tool

**Fig. 4:**
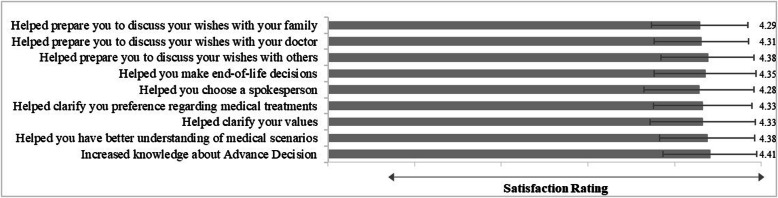
illustrates the feedback from participants’ experience of using VR video. Overall, the intervention was generally recognized by participants for its help in making decisions

Across the 9 items collecting feedback from participants’ experience (where 1 = strongly disagree, 5 = strongly agree), the highest rated item was “After the intervention, you thought that it increased your knowledge about advance decision” (4.41 ± 0.54). The lowest rated item was “After the intervention, you thought that it helped you choose a spokesperson” (4.28 ± 0.64). Overall, the intervention was generally recognized by participants for its help in making decision (See Fig. 4).

## Discussion

This study provides an innovative VR video approach to assist with ACP. To my knowledge, this study represents the first decision tool for ACP using VR technology.

When facing the possibility of meeting one of the five prescribed clinical conditions under the Act, approximately a quarter of participants were uncertain about the decision of whether to use or refuse LSTs before watching the VR video. However, for each treatment the percentage of people who were uncertain decreased to less than 20% after watching the VR video. Meanwhile, preference for not using these medical treatments had the opposite trend after watching the videos. The findings of our research were consistent with previous research that aimed at enriching patient understanding of worsening health states and informing their decision making with the use of a video decision tool [6]. According to our demographic data (Table 1), there were no significance differences between the different education levels, which means that this intervention can be adopted for people with different education and health literacy levels.

**Fig. 5:**
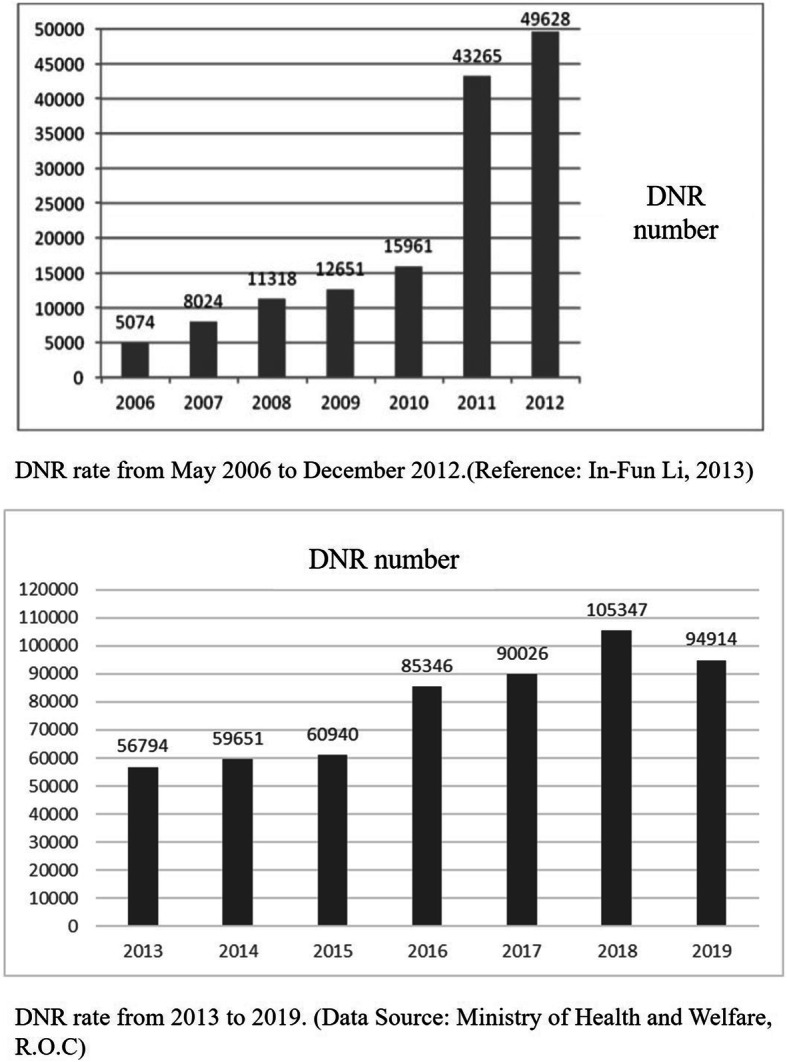
displays the DNR rate in Taiwan from 2006 to 2019 with a rapid growth of numbers since 2011 after the third amendment of The Hospice Palliative Care Ordinance of Taiwan (HPCOT)

End-of-life decision making has never been an easy task for people, especially in Asian cultures where it is taboo to talk about issues of death and palliative care. The Hospice Palliative Care Ordinance of Taiwan (HPCOT) was passed in 2000, with the aim to promote hospice palliative care and dying with dignity and to respect the wishes of patients with terminal illness and their right to personally decide about medical treatment. Although Asian culture still regards talking about death as taboo, HPCOT has had a significant impact on DNR rates (Fig. 5) [18]. The DNR numbers have a rapid growth since 2011 after the third amendment of HPCOT. Almost four-fifths of participants had heard about DNR orders. Before watching the VR video, the largest proportion of participants refused to use LST and CPR, which may be the result of people in Taiwan becoming more familiar with these terms due to HPCOT. Preference for not using these medical treatments significantly increased after viewing the VR video. The decrease in the number of participants who could not make a decision after watching the VR video indicates that our decision tool may help users make decisions. These results achieve the purpose of our research and are consistent with prior studies about video decision tools for ACP in patients with cancer [6].

The use of decision tools for medical decision making has been proved helpful in improving people’s knowledge regarding treatment options in previous studies [19]. The complexity of medical scenarios makes it relatively hard for people to imagine and clarify their real needs and make concrete choices about them. In response to this, we set a storyline for our video, and the scene changed from the ICU to a hospice home care setting from the first-person perspective. The way doctors spoke in the film followed the disease progression with the option of hospice care. According to the feedback, the VR video significantly helped equip users with a better understanding of medical scenarios. Developing a storyline with patient-centered ACP in a video has also been recognized as highly meaningful for patients and family members preparing for major surgery [20]. In addition, during development we collected professional opinions from medical staff as well as members of an interdisciplinary team including psychologists and senior social workers. Participants said that the VR video helped prepare them to discuss their wishes with their family and doctors.

Feedback collected from participants about this tool was positive, suggesting that it is a useful tool for preparing the users for ACP, insofar as study participants report being in agreement with how this VR video helped them 1) prepare to discuss issues with their family, medical team and others, 2) make end-of-life decisions 3) choose a spokesperson, 4) clarify their preferences for medical treatments and their values, and 5) have better understanding of medical scenarios and increased knowledge of advance decision.

## Limitations

Firstly, having no qualitative information collected to explore what participants thought these terms meant and further exploration is considered to confirm that the participants realized these terms correctly. Secondly, only one scenario was shown of the possible scenarios: “irreversible coma, permanent vegetative state, severe dementia, etc.” There is a strong degree of bias in influencing participants’ decisions by only showing one outcome (e.g., patient survives with reduced level of function). Providing only one type of scenario and one kind of outcome seems to have ethical considerations, but the VR video is produced and used as a decision tool, not for all of ACP and our consultation team discusses the pros and cons of any end-of-life care medical option to overcome this consideration. Thirdly, participants were mostly highly educated younger adults with no chronic or life-threatening illnesses, and their choices may be different from older participants with chronic life-limiting diseases. Fourthly, the emotional impact of viewing the video was not explored, but we can observe that the VR video induced the connection of memory about the tester’s end-of-life care experience about their close family members and we conducted a qualitative interview for these participants that we will explore further. Lastly, we cannot conclude that VR video is better than conventional videos from the results of this study, and the cost of making a VR film is higher than the cost of making a conventional video. However, according to the literature review, VR video can achieve better long-term retention of learned information [21]. The benefits of VR video need to be further explored to determine if VR video cost is mitigated by its effectiveness.

## Conclusion

Previous studies have revealed a gap between ADs and the real wishes of patients in palliative care. The traditional way for presenting ACP information to patients includes verbally communicating the given scenario between the medical team and patients [22]. Visual impact on decision-making has been mentioned in previous studies and the emotion, understanding, and motivation for discussing end-of-life care preferences are important issues in Taiwan’s society. ACP involves far more than merely establishing an AD for certain treatments; additional factors include family dynamics, emotional response, and the values of patients. These elements were emphasized in the VR film, but the real impact of VR video needs further study. Participants reported the highest satisfaction rating regarding the helpfulness of the VR video to increase knowledge about AD, which supports the fact that this study was effective in its purpose.

This is the first national study to explore the effectiveness of VR video as a decision tool in end-of-life care issues since the passing of the Act in Taiwan. This study revealed the VR video’s influence on the certainty in choosing and decreased preference of CPR, LST, antibiotics, blood transfusion and artificial nutrition and hydration. This decision tool also proved to be an effective tool for clarifying values and helping figure out and discussing end-of-life care preferences with others. In response to the implementing of the Act, we recommend this decision tool to promote this Act as well as preparing users for ACP.

## Data Availability

The datasets used and/or analyzed during the current study are available from the corresponding author on reasonable request.

## Abbreviations

ACP: Advance care planning
AD: Advance decision
VR: Virtual reality
DNR: Do not resuscitate
CPR: Cardiopulmonary resuscitation
COPD: Chronic obstructive pulmonary disease
LST: Life-sustaining treatment
